# Inheritance of esters and other volatile compounds responsible for the fruity aroma in strawberry

**DOI:** 10.3389/fpls.2022.959155

**Published:** 2022-08-12

**Authors:** Pol Rey-Serra, Mourad Mnejja, Amparo Monfort

**Affiliations:** ^1^Centre for Research in Agricultural Genomics (CRAG), CSIC-IRTA-UAB-UB, Barcelona, Spain; ^2^Institut de Recerca i Tecnologia Agroalimentàries (IRTA), Barcelona, Spain

**Keywords:** *Fragaria* × *ananassa*, aroma, QTL, key volatile compounds, marker-assisted breeding

## Abstract

Cultivated strawberry, *Fragaria*  ×  *ananassa*, has a complex aroma due to the presence of more than 350 volatile organic compounds (VOCs). However, a mixture of only 19 compounds, called Key Volatile Compounds (KVC), can impart the main strawberry aroma. The octoploid nature of the cultivated strawberry species (2*n* = 8*x* = 56) adds complexity to the heritance of the accumulation of the volatiles responsible for aroma. An F1 population cross between two breeding parental lines, FC50 and FD54, was phenotyped for aroma by SPME GCMS during six harvests. A total of 58 compounds were identified: 33 esters, nine terpenes, seven aldehydes, four lactones, two furans, one acid, one alkane and one alcohol, of which 16 were KVCs. A total of 179 QTLs were found, and 85 of these were detected in at least three harvests, of which 50 QTLs were considered major (LOD > 4.0) and detected in five or six analyzed harvests. Several clusters of ester QTLs associated with fruity aroma were discovered, such as QTLs for esters that share hexanoate group that were mapped in LG4A (*Hexanoate_4A*), those that share acetate and octyl groups in LG6A (*Acetate_6A* and *Octyl_6A*) or those with the same methyl group in LG7B (*Methyl_7B*). Different terpene QTLs associated with floral aroma appear grouped in a cluster in LG3C (*Terpene_3C*). Some of these clusters of QTLs were validated in a second F2 population, a cross of “Camarosa” and “Dover,” that was also phenotyped for three years. Selected SNPs from floral and fruity aroma QTLs were tested in a third population, which will most likely be useful for marker-assisted breeding (MAB).

## Introduction

Strawberries are one of the most consumed berries worldwide. During the last decades, strawberry breeding programs had been focused principally on fruit quality and disease resistance. However, consumers are demanding better tasting fruits (Folta and Klee, 2016) and for that reason, quality traits such as volatiles and polyphenolic content has gained importance in breeding programs. Volatile compounds, which increase during fruit ripening, are perceived as a healthy nutrient label (Goff and Klee, 2006).

In strawberries a total of 979 volatile organic compounds (VOCs) were identified (Ulrich et al., 2018), but humans perceive only a few of them which contribute to the strawberry aroma (Schieberle and Hofmann, 1997; Ulrich et al., 1997; Nuzzi et al., 2008; Urrutia et al., 2017). The large variability in strawberry volatile compounds can be explained by genetics, maturity stage and postharvest factors (Schwieterman et al., 2014). Woodland strawberry has a particular flavor and a more intense flavor than cultivated strawberry. Recent studies have shown that only a few VOCs correlate with human preferences and these enhance sweetness perception (Schwieterman et al., 2014; Fan et al., 2021).

Fatty acids are the major precursor of aromatic compounds, such as straight-chain acids, alcohols, aldehydes, esters, ketones and lactones. Esters, the most abundant chemical family in strawberry fruits, are produced by the esterification of alcohols, dehydrogenated by alcohol dehydrogenase (ADH), and acyl-CoA catalysed by the alcohol acyl transferases (AAT). In this last step, strawberry alcohol acyltransferase (SAAT) shows a wide range of substrate affinity (Aharoni et al., 2000; Beekwilder et al., 2004). Aldehydes and alcohols decrease during ripening at the same rate as the transcription level of AAT is increased in apricot (Gonzalez-Aguero et al., 2009) and during postharvest in peach (Zhang et al., 2010). Acyl-coA can be synthesised by β-oxidation or *via* lipoxygenases (LOX) being aldehydes and alcohol intermediates.

Branched and aromatic ester compounds are synthesised through amino acid metabolism. In strawberry, two eugenol synthases (*FaEGS1* and *FaEGS2*) are involved in the biosynthesis of eugenol and both are controlled by an R2R3 MYB transcription factor (*FaEOBII*; Aragüez et al., 2013; Medina-Puche et al., 2015). An anthranilate acid methyl transferase (FaAAMT) controls the last step of methyl anthranilate synthesis (Pillet et al., 2017). However, this compound is only detected in few cultivars and in woodland strawberry (Ulrich et al., 1997; Schwieterman et al., 2014).

Furthermore, Z-3/E-2-hexenal isomerases are responsible for converting Z-3-hexenal to E-2-hexenal as described in cucumber (Spyropoulou et al., 2017). Different studies reported that an omega-6 fatty acid desaturase (FaFAD1) is necessary for the lactone γ-decalactone biosynthesis (Chambers et al., 2014; Sanchez-Sevilla et al., 2014).

Furanones can also produce volatile compounds such as mesifurane and furaneol. A quinone oxidoreductase (FaQR) is required for furaneol biosynthesis (Raab et al., 2006) and an O-methyltransferases (FaOMT) methylate furaneol to produce mesifurane (Wein et al., 2002).

Finally, terpene compounds are also highly accumulated in strawberry fruits. Terpenes are derived from isopentenyl pyrophosphate (IPP) and dimethylallyl pyrophosphate (DMAPP) which can produce both monoterpenes and sesquiterpenes, independently. In strawberry, nerolidol synthase (FaNES1) can synthesise the monoterpene linalool and the sesquiterpene nerolidol (Aharoni et al., 2004). FaNES1 is present only in cultivated but not in wild strawberries (Aharoni et al., 2004). Moreover, FaNES1 has been widely screened in cultivated, wild octoploid and diploid accessions, reporting that only octoploid strawberries have the functional allele (Chambers et al., 2012). On the other hand, some compounds, such as myrtenol, play an important part in the aroma of woodland strawberry, and are not present in cultivated strawberry (Aharoni et al., 2004; Ulrich et al., 2007).

Among all the VOCs identified, 19 volatile compounds have been described as those most relevant in strawberries as aroma representatives and are considered as key volatile compounds (KVCs) in wild strawberry (Urrutia et al., 2017). Their importance lies in overcoming the threshold of odour perception in humans, and the combination of these 19 compounds produces the characteristic strawberry aroma (Schieberle and Hofmann, 1997; Ulrich et al., 1997, 2007). These KVCs are composed by 12 esters, two aldehydes, two furans, two terpenes and one lactone. Ester volatile compounds, such as butyl acetate, hexyl acetate, methyl butanoate, ethyl butanoate, butyl butanoate, methyl hexanoate or ethyl hexanoate, deliver fruity and sweet notes. Other esters such as methyl cinnamate providing spicy notes, myrtenyl acetate conferring herbaceous notes and methyl anthranilate that provides strawberry-like flavor are only present in few cultivars. The γ-decalactone is a lactone that provides a peach aroma. Aldehydes, such as E-2-hexenal and Z-3-hexenal and their ester derivatives, E-2-hexenyl acetate and Z-3-hexenyl acetate, deliver herbaceous notes. Furans, mainly furaneol and mesifurane, contribute to the strawberry aroma with caramel notes. Finally, terpene compounds, such as linalool and nerolidol, provide a floral aroma (Schieberle and Hofmann, 1997; Ulrich et al., 1997, 2007; Jetti et al., 2007; Nuzzi et al., 2008; Olbricht et al., 2008; Schwieterman et al., 2014).

Strawberry has an allo-octoploid genome composed by four subgenomes, one related to *Fragaria vesca*, another to *Fragaria iinumae* with the last two subgenomes being under discussion (Edger et al., 2020; Liston et al., 2020). The release of the *F.* × *ananassa* genome (Edger et al., 2019), increased the genomic knowledge of the cultivated strawberry. To characterize trait heritance, several dense genetic maps were constructed using the IStraw90k (Bassil et al., 2015) and IStraw35k SNP arrays (Verma et al., 2017). Recently, new 850 and 50 k SNP arrays have been developed (Hardigan et al., 2020).

QTLs for volatile compounds have been detected in previous studies. Zorrilla-Fontanesi et al. (2011) screened an F1 population by GC–MS and mapped 70 QTLs for 48 different volatile compounds, but only 35 of them were found to be stable over time. A near-isogenic line (NIL) collection using *F. vesca*, as the recurrent parent, and *F. bucharica*, as the donor parent, revealed 14 QTLs for different key volatile compounds (Urrutia et al., 2017). Moreover, RNAseq analysis identified two genes involved in the accumulation of two volatile compounds (Chambers et al., 2014; Sanchez-Sevilla et al., 2014; Pillet et al., 2017).

The main goal of this study is to increase the knowledge of the genetic regions responsible for the complex aroma of cultivated strawberry fruits. We will do this with two populations (the F1 of “FC50” × “FD54,” and the F2 of “Camarosa” × “Dover”) for which high-density linkage maps are already available (Rey-Serra et al., 2021). The VOC diversity obtained by GC–MS was analyzed in the F1 population from six harvests over three consecutive years, where a set of major and stable QTLs were identified. These were validated using a second F2 population and the SNPs associated with aroma KVC QTLs were used to select these traits in a breeding population.

## Materials and methods

### Plant material

An F1 population (*N* = 70) derived from a cross between two breeding lines, “FC50,” a seedling from the cross between “Donna” and “Cigaline” and “FD54,” an offspring from the cross between cultivars “Darselect Bright” and “Candonga” (hereafter “FC50 × FD54”). “FC50” has a wild strawberry aroma, whereas “FD54” has a characteristic fruity aroma. At least six clonal runner plants per progeny or parental lines were grown using standard cultivation practices in the south-west of France (Le Barp, latitude: 44°67′N, longitude 0°73′W) for three successive years (2016–2018). Ripe fruits were collected from six harvests over 3 years. Harvests from the same year were collected in one to 2 weeks’ intervals in May and June on 26/05/2016, 06/06/2016, 29/05/2017, 07/06/2017, 30/05/2018 and 07/06/2018. Each year, the plants have been clonally replicated by runners. Grandparents were also cultivated for further analysis.

A cross between two commercial cultivars, “Dover” and “Camarosa” produced a hybrid “H-21” which was self-pollinated to obtain an F2 population with 117 progenies (hereafter “21AF”). “Dover” was selected for its good agronomic performance and disease resistance, “Camarosa” for its fruit qualities such as its high polyphenol content. At least two clones per progeny or parental line were grown using standard cultivation practices in the north-east of Spain (Caldes de Montbui, latitude: 41°36′N, longitude 2°10′E) for 3 years (2014, 2015, and 2018). Ripe fruits were picked up over several days in May and June 2014, 2015, and 2018 and in this F2 population the harvested fruits from the same year were considered as an individual harvest. Each year, clonal runner plants were replicated.

### Volatile compound analysis by GC–MS

Each sample consisted of a pool of three to five strawberry fruits from each genotype and harvest, frozen in N_2 (liq.)_, powdered with a coffee grinder and stored immediately at −80°C. To perform the GC–MS assay, 1 g of powdered strawberry fruit was weighted into a 10 ml screw cap headspace vial and 1 ml of saturated NaCl solution added together with 10 ppm of internal standard (3-hexanone; Sigma-Aldrich, MO, United States). A GC Sampler 80 (Agilent Technology, CA, United States) was used for incubation, extraction and desorption. Vials were first heated at 50°C during 10 min with agitation at 500 rpm, then SPME fibre (50/30 μm DVB/CAR/PDMS; Supelco, PA, United States) was exposed into the headspace vial for 30 min in the same conditions to extract the volatile compounds. The extracted volatiles were desorbed in the GC injection port at 250°C for 5 min in splitless mode. Volatile compounds were analyzed on a 7890A gas chromatograph coupled with a 5975C mass spectrometer (Agilent Technology, CA, United States). An HP-5MS UI GC column (30 m, 0.250 mm, 0.25 μm; J&W, CA, United States) and 1.2 ml/min constant helium flow was used for chromatographic separation. Oven conditions began at 40°C for 2 min, increasing by 5°C/min ramp until reaching a temperature of 250°C and maintaining that temperature for 5 min.

All profiles were analyzed using Enhanced ChemStation software (Agilent Technologies, CA, United States) and compared with mass spectra libraries, NIST08 and NIST11. In addition 19 key volatile compounds (KVCs) were identified by comparing the retention time and ion fragmentation with each respective commercial standard (Sigma-Aldrich, MO, USA), and the remaining compounds by comparing the non-isothermal Kovats retention index (KI). The KI (van den Dool and Kratz, 1963) is calculated using C7-C30 saturated standards (Sigma-Aldrich, MO, United States). Our results were compared with KI from NIST database.^1^ Only those peaks that could be clearly identified were used for further analysis (Supplementary Table 1). Identified compounds areas were integrated by selecting a specific ion and multiplying by a response factor to get the real compound area. Then these areas were normalized by the 3-hexanone internal standard peak from each sample.

### Data analysis

The statistical analysis of GC–MS data was performed using R v3.6.2 (RCoreTeam, 2019) with the Rstudio v1.2.5033 interface (RStudio, 2019). A violin plot was obtained using “ggplot2” R package (version 3.3.0) that visualise relative VOCs content distribution. and Pearson correlation between VOCs were calculated using “cor” function and visualised as cluster network analyses (CNA) using “qgraph” function from “qgraph” R package (version 1.6.5) and Pearson correlation between harvests and VOCs were visualised by “corrplot” R package (version 0.84). Heatmap representation of hierarchical cluster analysis was performed using “heatmap.2” function from “gplots” R package (version 3.0.3). Principal component analysis (PCA) was done with “fviz_pca_biplot” function from “factorextra” R package (version 1.0.7). Omega squared values (ω^2^) were derived from ANOVA residuals calculated with “aov” function and using the formula: (SSi − df_i_ * MS_err_) * (MS_t_ + MS_err_)^−1^. Boxplots were drawn using “ggplot2” package (version 3.3.0) and “ggsignif” function (version 0.6.0) to calculate the *t*-test significant level.

### Volatile QTL analysis

To analyse the genetic regions responsible for the VOCs variance, a reduced “FC50 × FD54” genetic map was constructed with 1,464 markers grouped in 28 LGs and spanning 2,273 cM. Likewise, we used a reduced genetic map from the F2 “Camarosa” × “Dover” population (“21AF”) with 1,457 markers grouped in 29 LGs and spanning 1808.05 cM (Rey-Serra et al., 2021).

VOC data was transformed with log_2_ and analyzed separately for each harvest in the MapQTL^®^6 software (van Ooijen, 2009) using the Interval Mapping method (IM) and nonparametric Kruskal–Wallis test (KW). Same criteria as Rey-Serra et al. (2021) was followed to classify QTLs. A QTL was determined with a minimum LOD score of 2.5 by IM and when detected in at least two harvests. The threshold to identify a major QTL was calculated by a permutation test and established at a LOD score of four. KW has been used to determine the most accurately linked marker. *R*^2^ determines the percentage of phenotypic variation expressed by the QTL. The MapChart2.2 software (Voorrips, 2002) was used to visualize each QTL in our genetic map with fill and color parameters that distinguish the stability and chemical family of each VOC, respectively. The QTL size was determined according to the overlapping 1-LOD confidence interval to define either side of the peak.

To identify the physical position of each marker, SNP probes were blasted to the *F.* × *ananassa* cv. “Camarosa” genome (Edger et al., 2019) as explained in Rey-Serra et al. (2021). Once the genome distances of the QTLs had been delimited, genes inside each QTL were analyzed by gene homology using NCBI, SwissProt, Tair10 and TrEMBL database obtained from https://www.rosaceae.org/species/fragaria_x_ananassa/genome_v1.0.a1 (Jung et al., 2019).

## Results

### Volatile compound composition

To identify the compound variability in the strawberry aroma, full ripe fruits from 68 plants of the “FC50 × FD54” population collected in six different harvests over three consecutive years (2016–2018) were analyzed by GC–MS. From hundreds of peaks present in strawberry aroma profile, 58 volatile organic compounds (VOCs) could be clearly identified (Table 1; Supplementary Table 2) including, 33 (57%) esters, nine (16%) terpenes, seven (12%) aldehydes, three (5%) lactones, two (3%) furans and four (7%) from compounds different to other families. Out of the 19 compounds considered as key volatile compounds (KVCs), 16 were identified (Table 1). Myrtenyl acetate and methyl cinnamate peaks were not present in this population and the Z-3-hexenal peak was almost undetectable.

**Table 1:** VOCs summary in “FC50 × FD54” population.

Volatile compound	Abb.	Type	FC50	FD54	FC50 × FD54	
			Mean ± SD	Mean ± SD	Mean ± SD	Range
**Butyl acetate** ^*^	BA	Ester	0.092 ± 0.043	0.118 ± 0.058	0.104 ± 0.075	0.007–0.528
Isopentyl acetate	IPA	Ester	0.042 ± 0.011	0.074 ± 0.061	0.065 ± 0.060	0.005–0.537
**Hexyl acetate** ^*^	HA	Ester	0.152 ± 0.062	0.461 ± 0.385	0.332 ± 0.534	0.013–6.350
Octyl acetate	OA	Ester	0.026 ± 0.042	0.129 ± 0.103	0.045 ± 0.099	0.002–0.953
**Methyl butanoate** ^*^	MB	Ester	2.938 ± 1.132	2.104 ± 0.605	3.507 ± 1.580	0.140–9.569
**Ethyl butanoate** ^*^	EB	Ester	2.724 ± 1.837	1.630 ± 0.886	3.598 ± 3.182	0.064–21.773
Isopropyl butanoate	IPB	Ester	0.411 ± 0.122	0.417 ± 0.253	0.443 ± 0.292	0.012–1.445
Methyl 3-methylbutanoate	M3MB	Ester	0.041 ± 0.013	**0.126 ± 0.064**	0.083 ± 0.060	0.011–0.438
Propyl butanoate	PB	Ester	0.056 ± 0.029	0.056 ± 0.033	0.065 ± 0.048	0.003–0.262
**Butyl butanoate** ^*^	BB	Ester	0.419 ± 0.205	0.511 ± 0.478	0.630 ± 0.705	0.002–4.076
Hexyl butanoate	HB	Ester	0.073 ± 0.040	0.418 ± 0.509	0.239 ± 0.399	0.003–4.972
Octyl butanoate	OB	Ester	0.031 ± 0.042	0.680 ± 0.718	0.380 ± 0.843	0.002–7.411
1-Methyloctyl butanoate	MOB	Ester	0.350 ± 0.690	0.040 ± 0.046	0.060 ± 0.226	0.001–3.183
Octyl 3-methylbutanoate	O3MB	Ester	0.004 ± 0.002	0.274 ± 0.275	0.046 ± 0.100	0.002–1.113
Methyl 4-methylpentanoate	M4MP	Ester	0.017 ± 0.012	0.017 ± 0.017	0.024 ± 0.024	0.002–0.167
**Methyl hexanoate** ^*^	MH	Ester	2.876 ± 1.078	2.961 ± 1.802	3.840 ± 2.258	0.028–12.808
**Ethyl hexanoate** ^*^	EH	Ester	1.592 ± 1.552	1.777 ± 1.478	3.231 ± 3.626	0.009–20.876
Isopropyl hexanoate	IPH	Ester	0.084 ± 0.030	0.116 ± 0.075	0.143 ± 0.145	0.002–1.186
Isoamyl hexanoate	IAH	Ester	0.014 ± 0.007	0.021 ± 0.016	0.045 ± 0.063	0.001–0.419
Hexyl hexanoate	HH	Ester	0.007 ± 0.003	0.101 ± 0.140	0.032 ± 0.048	0.003–0.372
Octyl hexanoate	OH	Ester	0.005 ± 0.002	0.202 ± 0.228	0.091 ± 0.248	0.002–2.833
Methyl E-2-hexenoate	M2H	Ester	0.100 ± 0.039	0.054 ± 0.035	0.069 ± 0.063	0.003–0.388
Ethyl 2-hexenoate	E2H	Ester	0.006 ± 0.004	0.010 ± 0.008	0.020 ± 0.031	0.002–0.381
Methyl octanoate	MO	Ester	0.062 ± 0.026	0.363 ± 0.310	0.206 ± 0.234	0.001–1.768
Ethyl octanoate	EO	Ester	0.007 ± 0.005	0.036 ± 0.024	0.067 ± 0.142	0.001–1.273
Methyl decanoate	MD	Ester	0.001 ± 0.000	**0.023 ± 0.011**	0.011 ± 0.013	0.001–0.097
Methyl benzene acetate	MBA	Ester	0.037 ± 0.011	**0.081 ± 0.033**	0.054 ± 0.031	0.007–0.266
Methyl nicotinate	MN	Ester	**0.038 ± 0.027**	0.002 ± 0.002	0.016 ± 0.028	0.001–0.236
Methyl salicylate	MS	Ester	0.090 ± 0.054	0.040 ± 0.032	0.090 ± 0.085	0.002–0.506
**Methyl anthranilate** ^*^	MA	Ester	**0.252 ± 0.252**	0.009 ± 0.008	0.065 ± 0.180	0.001–1.752
**Z-3-hexenyl acetate** ^*^	Z3HA	Ester	0.028 ± 0.024	0.039 ± 0.036	0.030 ± 0.019	0.004–0.134
**E-2-hexenyl acetate** ^*^	E2HA	Ester	0.069 ± 0.065	0.156 ± 0.192	0.166 ± 0.199	0.009–2.509
E-2-hexenyl butanoate	E2HB	Ester	0.032 ± 0.021	0.086 ± 0.159	0.105 ± 0.182	0.002–2.070
Hexanal	Hex	Aldehyde	0.144 ± 0.050	**0.526 ± 0.266**	0.353 ± 0.220	0.064–1.272
Heptanal	Hep	Aldehyde	0.026 ± 0.001	**0.042 ± 0.008**	0.043 ± 0.018	0.017–0.139
Nonanal	Non	Aldehyde	0.230 ± 0.051	0.166 ± 0.103	0.209 ± 0.153	0.007–1.070
Decanal	Dec	Aldehyde	0.011 ± 0.007	0.043 ± 0.062	0.021 ± 0.023	0.003–0.315
**E-2-Hexenal** ^*^	E2Hal	Aldehyde	1.990 ± 0.521	2.290 ± 1.430	1.865 ± 1.434	0.064–9.109
Z-2-Heptenal	Z2H	Aldehyde	0.024 ± 0.002	**0.048 ± 0.019**	0.048 ± 0.018	0.014–0.121
Benzaldehyde	B	Aldehyde	0.154 ± 0.122	0.187 ± 0.150	0.200 ± 0.160	0.007–1.260
Dodecane	Dode	Alkane	0.039 ± 0.042	**0.011 ± 0.010**	0.029 ± 0.056	0.001–1.017
Octanoic acid	Oac	Acid	0.074 ± 0.072	0.203 ± 0.209	0.176 ± 0.312	0.003–2.936
Octanol	Ool	Alcohol	0.082 ± 0.063	0.071 ± 0.049	0.068 ± 0.053	0.009–0.386
2-Heptanone	Hep2	Ketone	0.156 ± 0.083	0.291 ± 0.268	0.169 ± 0.146	0.010–1.293
γ-octalactone	GO	Lactone	0.004 ± 0.004	**0.043 ± 0.030**	0.023 ± 0.021	0.001–0.180
**γ-decalactone** ^*^	GD	Lactone	0.046 ± 0.063	**4.046 ± 2.068**	2.720 ± 1.901	0.001–14.162
γ-dodecalactone	GDo	Lactone	0.124 ± 0.189	0.292 ± 0.398	0.195 ± 0.288	0.001–1.591
**Mesifurane** ^*^	M	Furan	3.375 ± 1.271	1.828 ± 1.410	2.419 ± 2.242	0.023–13.937
**Furaneol** ^*^	F	Furan	0.280 ± 0.087	0.111 ± 0.079	0.118 ± 0.144	0.006–1.018
β-Pinene	BP	Terpene	0.096 ± 0.079	**0.278 ± 0.127**	0.199 ± 0.162	0.001–1.002
D-Limonene	DL	Terpene	0.035 ± 0.030	**0.115 ± 0.054**	0.080 ± 0.063	0.002–0.415
α-Terpineol	AT	Terpene	0.076 ± 0.057	**0.375 ± 0.186**	0.171 ± 0.162	0.002–1.242
**Linalool** ^*^	L	Terpene	0.854 ± 0.395	**2.421 ± 0.691**	1.488 ± 0.919	0.010–5.554
Geraniol	G	Terpene	0.002 ± 0.001	**0.008 ± 0.006**	0.004 ± 0.005	0.000–0.034
β-Farnesene	BF	Terpene	0.161 ± 0.182	0.414 ± 0.338	0.296 ± 0.278	0.003–1.460
α-Curcumene	AC	Terpene	0.032 ± 0.044	0.063 ± 0.073	0.052 ± 0.064	0.001–0.403
α-Farnesene	AF	Terpene	0.096 ± 0.097	0.292 ± 0.217	0.203 ± 0.191	0.003–1.268
**Nerolidol** ^*^	N	Terpene	0.382 ± 0.300	0.962 ± 0.670	0.603 ± 0.529	0.003–2.706

Volatile compound, its abbreviation and compound family. Relative average and standard deviation (SD) of 58 VOCs content detected in parental lines “FC50” and “FD54” and relative average, standard deviation and range for their F1 progenies. Volatile compound in bold and ^*^ mark KVCs, Values in bold indicate the highest significant (*p* < 0.05) value in parental lines.

The compounds that showed the highest peaks were methyl butanoate, ethyl butanoate, butyl butanoate, methyl hexanoate, ethyl hexanoate, E-2-hexenal, mesifurane, γ-decalactone and linalool which are KVCs (Table 1; Supplementary Figure 1). Despite the fact that VOC content differed among harvests, the “FD54” parental line displayed a significantly higher (*p*-value <0.05) concentration of most VOCs compared to the “FC50” parental line, such as some esters (methyl 3-methylbutanoate, methyl decanoate and methyl benzeneacetate), aldehydes (hexanal, heptanal and Z-2-heptanal), lactones (γ-octalactone and γ-decalactone) and terpenes (β-pinene, D-limonene, α-terpineol, linalool and geraniol), whereas the concentrations of methyl anthranilate and methyl nicotinate were significantly higher in the “FC50” parental line than in “FC54.” In addition, “FC50 × FD54” progeny showed greater variation for each VOC than the parental lines (Supplementary Figure 1).

VOC distribution in each harvest was checked by a Shapiro–Wilk test which revealed that few compounds (methyl butanoate, methyl hexanoate, hexanal, E-2-hexenal, Z-2-heptanal and γ-decalactone) were normally distributed. However, when the VOC content was transformed to log_2,_ the majority (42/58) presented a normal distribution in at least three harvests (Supplementary Table 3).

### Volatile correlation analysis

**Figure 1:**
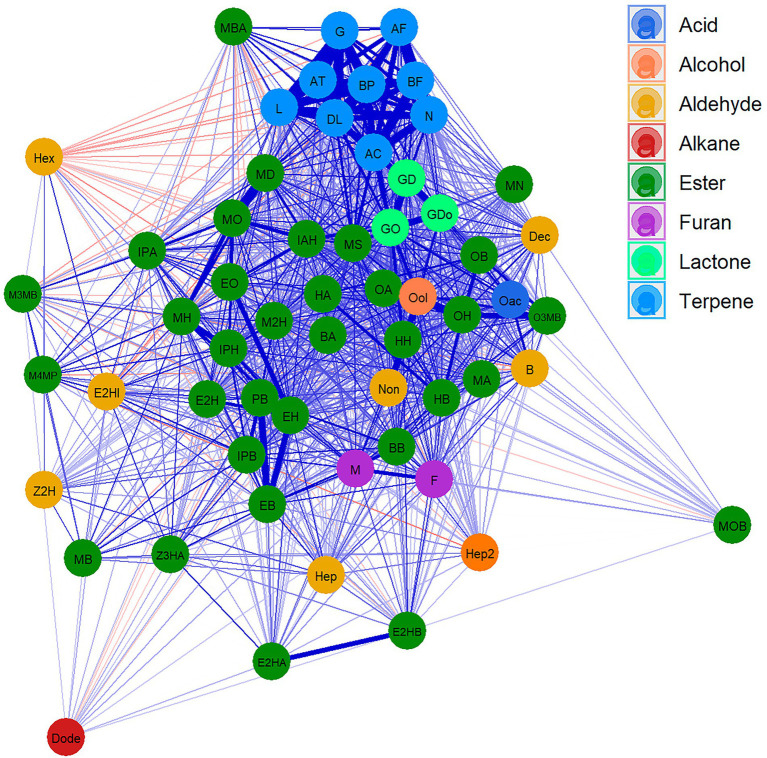
Cluster network analysis (CNA) for VOCs in “FC50 × FD54.” Compounds are represented as nodes and colored according to the chemical families. Positive (blue) and negative (red) correlation are represented as link between compounds, the widest links being the strongest correlation. Showing correlations with *p* value <0.05. For the correspondences between the full name and their abbreviation names consult Table 1.

A Cluster network analysis (CNA) was drawn to visualise the relationships between different VOCs (Figure 1; Supplementary Table 4). This analysis clearly clustered the different terpene compounds that show a strong correlation and separates them from the rest of the VOCs. Esters, which are the most abundant compounds, were shown to be highly correlated with each other, while branched esters display less correlation with other compounds. Lactones showed a high correlation with each other and with few esters. Similarly, furan compounds revealed a high correlation with each other and with hexyl butanoate. Only the aldehyde family presented a low correlation between its components. Indeed, few weak negative correlations were detected.

To further understand the relationship between compounds in different harvests, Pearson correlation between VOCs, independently of the harvest, were calculated (Supplementary Table 5). The results showed that ester compounds sharing the same acyl-CoA group were highly correlated (0.43–0.90 correlation range between VOCs and harvests), such as propyl butanoate with ethyl butanoate, isopropyl butanoate with butyl butanoate, and methyl hexanoate with propyl hexanoate and with isoamyl hexanoate. Similarly, esters having the same alcohol group showed high correlations (between 0.30 and 0.97), such as methyl hexanoate with methyl octanoate and with methyl decanoate, ethyl hexanoate with ethyl 2-hexenoate, with ethyl octanoate and with ethyl butanoate, methyl octanoate with methyl decanoate, hexyl butanoate with hexyl acetate, and octyl acetate with octyl 3-methylbutanoate and with octyl hexanoate (Supplementary Figure 2).

Lactones also presented high correlations, the highest being between γ-dodecalactone and γ-decalactone (0.52–0.83). Likewise, furans showed a greater correlation between each other than with other compounds (Supplementary Figure 2).

The most stable correlations across the different harvests were detected between terpene compounds. Indeed, the minimal correlation between monoterpenes was 0.74, while for sesquiterpenes it was 0.77. However, the minimal correlation between monoterpene and sesquiterpene compounds was quite low (0.32). Notably, linalool showed the highest correlations among all terpene compounds ranging from 0.60 to 0.99 (Supplementary Figure 2).

Moreover, high correlations between compounds belonging to different families were also observed. For instance, some butanoate related esters and furans presented a high correlation between each other. Likewise, some lactones with ester compounds and some terpenes with compounds from other families with a maximum correlation of 0.76 (Supplementary Figure 2).

**Figure 2:**
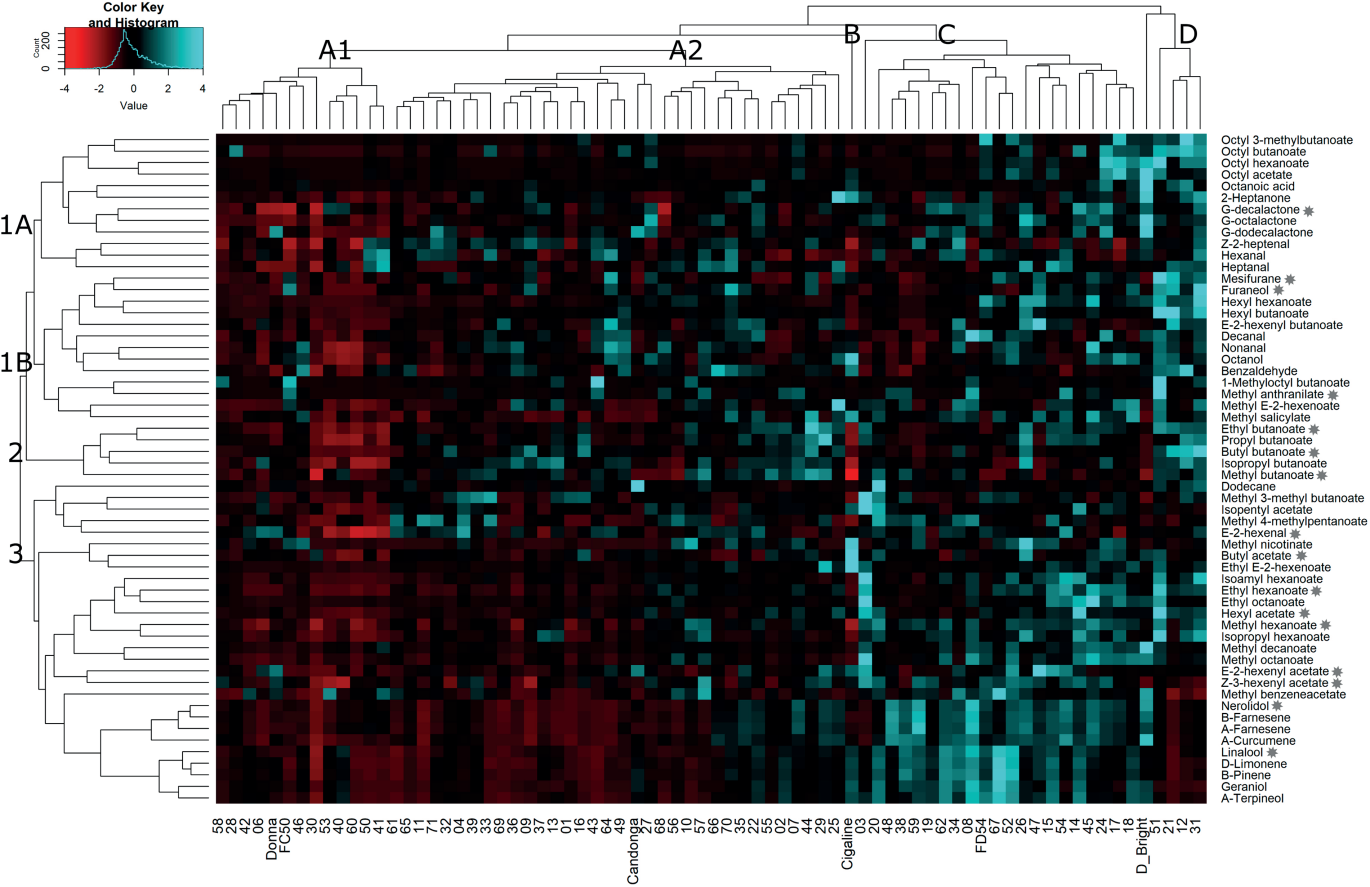
Heatmap representation of hierarchical clustering analyses (HCA) in VOCs in the “FC50 × FD54” population. Clusters are named by capital letters for individuals and numbers for VOCs. Stars indicate KVCs.

To better understand the relationship between VOCs and progenies, parental lines (“FC50” and “FD54”) and grandparental lines (“Donna,” “Cigaline,” “Darselect Bright” and “Candonga”), a hierarchical cluster analysis (HCA) using the average of six harvests was performed. A heatmap representation of the HCA shows three main compounds and four individual clusters (Figure 2). Compound cluster 1 agglomerated 43% of the VOCs, which is divided into two subclusters, 1A and 1B. Compound cluster 1A, contained 21% of the VOCs and grouped esters derived from octyl-CoA and lactones. Compound cluster 1B, comprised 22% of the VOCs and clustered aldehydes and hexyl-CoA esters. Compound cluster 2, the smallest one, grouped 9% of the VOCs, being all butanoate derived esters. Finally, compound cluster 3 agglutinated 48% of the VOCs that join terpenes, hexanoate derived esters and branched ester, where the latter is the furthest from the others.

The hierarchical cluster analysis defines four groups of genotypes which could be interesting for breeding purposes (Figure 2). Most of the lines (64%), were grouped together in a big cluster A, which is separated into two subclusters, A1 and A2. Cluster A1 included 18% of the individuals such as “FC50” and “Donna” and was characterized by its relatively low VOC content. Cluster A2, the largest group, comprised 46% of the individuals such as “Candonga,” and was distinguished by its high content of several VOCs, but also by its low terpene content. Cluster B, represented by a unique sample, “Cigaline,” was characterized by its high content of octanol, methyl nicotinate, butyl acetate and ethyl E-2-hexanoate. Cluster C corresponded to 28% of the individuals that includes “FC54.” This cluster was characterized by showing a high terpene and several ester contents. The last group, cluster D with only 5 individuals (7%), includes “Darselect Bright,” and differs by its high content of almost all VOCs except terpenes (Figure 2).

### Analysis of the environmental effect on VOCs

In response to varying environmental conditions, plants could alter the biosynthesis of some VOCs in different ways. We assumed that each harvest, cultivated in the open field, to be under different environmental conditions and therefore, its VOC accumulation would be affected. To figure out how different environmental conditions might affect VOC content, a Principal component analysis (PCA) was performed. The first three dimensions explained 26.5% (Dim1), 11.9% (Dim2) and 8.2% (Dim3) of the VOC variance. Each harvest partially overlapped the others in both PCA plots. The closest harvests were from the same year, but slight differences arose between years, the most divergent being 2018 (Supplementary Figure 3).

Regarding the contribution of VOCs to each PCA plot, the plot of Dim1 and Dim2 showed that terpene, lactone and aldehyde compounds were clustered together and esters such as the octyl group and hexanoate related esters were forming a small and dense group. Other ester compounds were scattered (Supplementary Figure 3A). However, no clear pattern was found when plotting together the Dim 1 and Dim 3 (Supplementary Figure 3B).

To dissect the variance caused by the genotype (G), the environment (E) or their interaction (GxE) regarding the VOC accumulation, the significance of each factor and trait was calculated and quantified (Supplementary Table 6). The analysis of variance (ANOVA), taking into account the genotype, year and interaction between both (G + E + GxE), revealed 50 VOCs with significant genetic differences (*p*-value<0.05), 48 VOCs depending significantly on the environmental effect and only 18 VOCs exhibiting a significant effect of GxE interaction.

In general, the genotypic factor was the main contributor to the observed variance over environment and GxE. At least 25% of the observed variance was explained by the G factor in 26 VOCs. This genetic variance was particularly high in linalool, nerolidol and octyl 3-methyl butanoate, explaining about 40% of their variance. In contrast, a high influence of the E factor was detected in some VOCs, explaining more than 30% of their total observed variance, such as methyl salicylate, nonanal, octanol and γ-nonalactone. The GxE effect never exceeded 20% of the total observed variance in any VOCs. The part of the VOC variability that cannot be attributed to any of the above factors, was named “error” (Supplementary Table 6).

### Key volatile compounds in the “21AF” population

Fully ripe fruits from a second population (“21AF”) were similarly analyzed by GC–MS for three harvests (2014, 2015, and 2018) to measure their KVCs (Supplementary Table 7). In this population, methyl anthranilate and furaneol were not detected. The distribution analysis shows that the KVCs in each year were normal distributed and overlapped on violin plot representation (Supplementary Figure 4A; Supplementary Table 8). As described in F1 population, high correlations were detected between ester compounds that share the same acyl-CoA group (0.49–0.79 in range), such as between butyl acetate and hexyl acetate, or the same alcohol group, such as between methyl butanoate and methyl hexanoate or between ethyl butanoate and ethyl hexanoate. Also, high correlations were found between other ester compounds, such as between hexyl acetate and butyl butanoate or between hexyl acetate and ethyl hexanoate. In addition to esters, two terpene compounds, linalool and nerolidol, also exhibited high correlation between each other ranging between 0.60 and 0.71 according to the year (Supplementary Figure 4B). The performed hierarchical cluster analysis classified the genotypes by groups in relation to their KVC accumulation. (Supplementary Figure 4C). The same overlapping behavior, as for the F1 population, was observed in the PCA plots when comparing all KVCs from different years together (Supplementary Figure 4D).

### Volatile QTL detection

**Figure 3:**
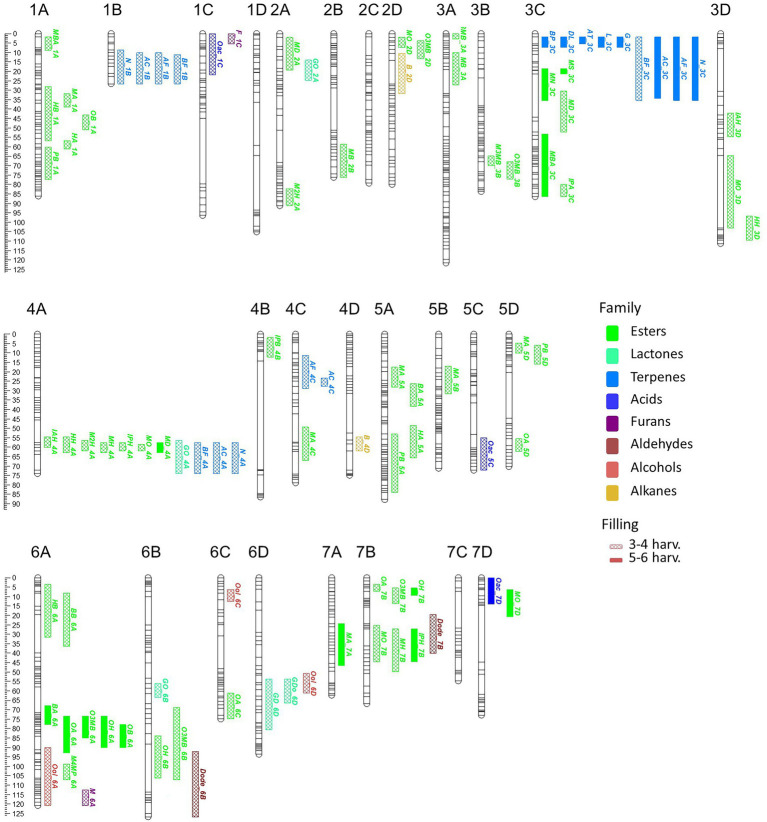
VOC_QTL location in the “FC50 × FD54” genetic map. Box size correspond to the 1-LOD confidence interval. Stability is represented by box filling degree: consistent in five or six harvests (filled) and detected in three or four harvests (semi-filled). Chemical family compounds are differentiated by colors: acids (light blue), alcohols (red), aldehydes (yellow), alkanes (dark red), esters (green), furans (purple), lactones (turquoise) and terpenes (dark blue).

A total of 58 VOCs were characterized by GC–MS in the “FC50 × FD54” population, of which 16 were considered KVCs. By the genetic analyses, a total of 179 QTLs were discovered, exhibiting a LOD > 2.5 in at least two different harvests, for 55 different VOCs (Supplementary Table 9). From these, 94 QTLs were detected in more than three harvests and 22 in five to six harvests (Figure 3). Up to 50 QTLs were considered major (LOD > 4.0) and described with more detail in Table 2. A total of 44 QTLs were mapped for 14 KVCs, of which 11 were major. Only two KVCs, ethyl butanoate and E-2-hexenyl acetate, did not show any significant QTL.

**Table 2:** VOC_QTLs detected in “FC50 × FD54” and “21AF” population.

QTL ID	KVC	Har.	LOD	*R* ^2^	Genetic map				*F.* × *ananassa* genome	
					LG	QTL boundaries (cM)		QTL interval (cM)	QTL interval (pb)	Nb. genes
FC50 × FD54										
BA_6A	X	5	5.19	35.2	6A	68.03	77.92	9.89	5,541,008	753
OA_6A		6	14.41	69.4	6A	73.39	92.94	19.55	11,087,539	1,652
MB_2B	X	4	4.07	28.5	2B	58.73	76.17	17.44	6,021,672	1,021
IPB_4B		3	5.53	37.6	4B	1.86	12.14	10.29	152,931	13
M3MB_3B		4	6.52	41.5	3B	64.92	69.99	5.07	5,783,583	653
PB_5A		4	4.13	29.2	5A	53.10	84.11	31.01	16,168,702	2,154
PB_5D		3	4.91	33.2	5D	5.91	15.94	10.03	6,955,239	1,108
HB_1A		4	5.06	35.1	1A	28.15	56.67	28.52	6,127,553	1,037
HB_6A		4	4.64	32.7	6A	3.65	31.50	27.85	6,369,285	1,096
OB_1A		3	5.72	38.6	1A	43.14	50.98	7.84	2,217,834	339
OB_6A		6	11.26	61.7	6A	77.92	90.13	12.21	6,023,570	830
O3MB_2D		4	4.51	31.9	2D	3.71	13.37	9.67	6,336,099	653
O3MB_6A		6	14.4	70.7	6A	73.39	85.13	11.74	6,023,570	830
MH_4A	X	4	6.91	44.5	4A	57.48	62.90	5.42	7,402,050	1,233
MH_7B	X	3	5.49	36.3	7B	27.13	49.80	22.67	10,345,159	1,754
IPH_4A		4	4.51	31.9	4A	57.48	61.90	4.42	7,402,050	1,233
IPH_7B		6	9.08	53.9	7B	27.13	44.57	17.44	4,953,047	765
IAH_4A		4	5.25	36.1	4A	54.49	60.08	5.59	7,402,050	1,233
HH_4A		3	4.48	31.8	4A	54.49	62.90	8.41	7,402,050	1,233
OH_6A		6	12.59	65.2	6A	73.39	90.13	16.74	6,023,570	830
M2H_4A		3	4.32	30.8	4A	56.49	61.90	5.41	7,402,050	1,233
MO_4A		4	7.22	46	4A	58.48	61.90	3.42	7,402,050	1,233
MO_7D		5	4.03	28.6	7D	6.36	20.70	14.33	7,059,468	849
MD_4A		6	9.98	57.3	4A	57.48	62.90	5.42	7,402,050	1,233
MBA_1A		3	4.37	31.1	1A	1.82	8.85	7.03	1,881,958	382
MBA_3C		6	4.45	31.1	3C	53.25	86.54	33.29	14,891,344	1,682
MN_3C		6	14.93	72	3C	18.55	35.35	16.80	2,519,086	358
MS_3C		6	13.63	68	3C	18.55	21.35	2.80	2,519,086	358
MA_1A	X	3	5.07	34.6	1A	31.87	38.92	7.06	2,839,656	526
MA_5D	X	3	4.16	29	5D	4.85	10.09	5.24	5,079,981	809
MA_7A	X	6	7.3	46.4	7A	24.26	46.52	22.26	16,373,010	2,767
Dode_6B		3	4.15	28.9	6B	92.16	126.82	34.65	5,991,681	1,078
Dode_7B		4	6.85	44.2	7B	19.56	40.22	20.66	2,871,386	395
Oac_1C		3	4.85	33.9	1C	0.00	21.93	21.93	3,554,988	667
Oac_7D		5	6.25	41.3	7D	0.00	14.05	14.05	11,737,287	1,243
Ool_6D		3	4.54	31.6	6D	50.90	61.34	10.43	4,473,829	502
GDo_6D		4	4.06	28.8	6D	53.81	66.63	12.82	9,105,053	1,010
F_1C	X	3	4.74	33.2	1C	0.00	5.52	5.52	1,505,596	283
BP_3C		6	7.15	45.6	3C	1.82	7.43	5.62	954,222	171
DL_3C		5	7.32	45.2	3C	1.82	7.43	5.62	954,222	171
AT_3C		5	6.2	39.9	3C	1.82	5.52	3.71	954,222	171
L_3C	X	5	7.06	44	3C	1.82	7.43	5.62	954,222	171
G_3C		6	7.01	43.8	3C	1.82	7.43	5.62	954,222	171
BF_3C		4	7.23	44.8	3C	1.82	35.35	33.53	6,795,619	1,077
BF_4A		3	5	34.7	4A	57.48	73.96	16.48	11,331,026	1,980
AC_3C		5	7.25	44.9	3C	1.82	34.35	32.53	6,795,619	1,077
AC_4A		4	4.94	34.4	4A	57.48	73.96	16.48	11,331,026	1,980
AF_3C		5	7.31	45.2	3C	1.82	35.35	33.53	6,795,619	1,077
N_3C	X	5	7.17	44.6	3C	1.82	35.35	33.53	6,795,619	1,077
N_4A	X	3	4.76	33.4	4A	57.48	73.96	16.48	11,331,026	1,980
21AF										
BA_6A	X	3	5.08	21.5	6A	59.39	66.61	7.22	3,623,024	481
HA_6A	X	3	5.70	22.1	6A	60.99	70.18	9.19	1,739,375	237
MB_1A	X	3	4.58	17.3	1A	24.32	31.79	7.46	3,002,207	481
MB_7B	X	3	9.13	32.1	7B	21.18	26.40	5.22	594,943	93
MH_1A	X	3	4.37	17.4	1A	21.25	37.88	16.63	3,838,906	635
MH_7B	X	3	10.57	37.1	7B	21.18	26.40	5.22	594,943	93
GD_3B	X	3	7.61	30.0	3B	72.76	79.29	6.53	2,441,208	368
L_3C	X	3	7.61	27.3	3C	0.00	14.02	14.02	31,373	9
N_3C	X	3	9.61	34.4	3C	0.00	0.86	0.86	31,373	9

List of major (LOD > 4) and stable (more than 2 harvests) VOC_QTLs ordered by compounds. Maximum values of LOD score, *R*^2^ explain % of phenotypic variance, QTL interval determined by 1-LOD confidence interval, QTL position in *F.* × *ananassa* genome and number of genes in each region.

Focusing on KVC_QTLs of the ester family, two butyl acetate QTLs were mapped: BA_5A, with *R*^2^ ≤ 27.0, and BA_6A, a major QTL detected in five of the six analyzed harvests with *R*^2^ ≤ 35.2. Significant QTLs for hexyl acetate were detected and the most important ones are HA_1A and HA_5A with *R*^2^ ≤ 24.6. For methyl butanoate, different QTLs were discovered such as MB_2B, a major QTL with *R*^2^ ≤ 28.5 and MB_3A with *R*^2^ ≤ 20. For butyl butanoate, a single QTL was mapped in LG6A with *R*^2^ ≤ 27.4. Two major QTLs were detected for methyl hexanoate that are MH_4A present in four of the six harvests with *R*^2^ ≤ 44.5 and MH_7B observed in half of the harvests with *R*^2^ ≤ 36.3. Six different QTLs were detected for methyl anthranilate, of which two were constant in three of the six harvests that are MA_1A, which explained *R*^2^ ≤ 34.6 and MA_5D with *R*^2^ ≤ 29.0. A third interesting major QTL for MA was consistent in all harvests, which in LG7A explained a range of *R*^2^ between 22.7% and 46.4% of the phenotypic variance (Supplementary Table 9).

For the terpene family, two major QTLs were detected in five of the six harvests and mapped together with the two most important terpenes, linalool and nerolidol, in LG3C (L_3C and N_3C) with *R*^2^ ≤ 44.6 of both variances. For nerolidol, another major QTL was identified N_4A, with *R*^2^ ≤ 33.4 (Table 2).

With respect to lactone family, one QTL was discovered for γ-decalactone in LG6D *R*^2^ ≤ 25.5. For furans, two QTLs were constant in three of the six harvests, F_1C a major QTL for furaneol, with *R*^2^ ≤ 33.2, and M_6A a QTL for mesifurane with *R*^2^ ≤ 25.1.

Looking at map distribution, QTLs for ester compounds were present in almost all linkage groups. Major QTLs of octyl ester compounds were mapped in LG6A, LG6B and LG7B, being the QTL in LG6A the most significant and stable. In LG1A, few ester QTLs were mapped closely to the OB_1A QTL, such as HA_1A, HB_1A, PB_1A and MA_1A. Several QTLs mainly related to hexanoate but also to octanoate and decanoate (MH_4A, IAH_4A, IPH_4A, HH_4A, M2H_4A, MO_4A and MD_4A) were mapped at the end of LG4A. Moreover, QTLs of MH_7B, IPH_7B and MO_7B were detected in LG7B and were mapped close to some octyl ester QTLs (Figure 3; Supplementary Table 9).

All identified terpenes showed QTLs that were major and consistent in at least five of the six harvests (L_3C, BP_3C, DL_3C, AT_3C, G_3C, N_3C, AF_3C, BF_3C and AC_3C). All of them shared the same region at the beginning of LG3C. In addition to these major QTLs, stable QTLs for monoterpene compounds were detected in LG3A and for sesquiterpenes in LG1B, LG4A and LG4C (Figure 3; Supplementary Table 9). The QTLs for lactone compounds were found in few LGs, and the major QTL GDo_6D was co-located in the lower part of the LG6D with GD_6D.

The QTL analysis in the 21AF population showed a total of 51 QTLs for 14 KVCs, but only nine of these were major and consistent in all harvests. Six of these QTLs were for ester compounds, one for lactones and two for terpenes (Table 2; Supplementary Table 9). Concerning the ester QTLs, methyl butanoate and methyl hexanoate QTLs were mapped in the same region in LG1A (MB_1A and MH_1A) and in LG7B (MB_7B and MH_7B), while two other ester compounds, butyl acetate and hexyl acetate, were located in LG6A (BA_6A and HA_6A). For terpenes, linalool and nerolidol QTLs were located at the beginning of LG3C, and for lactones, the γ-decalactone QTL was mapped at the end of LG3B (Table 2).

### QTL validation in the two populations

In order to compare and validate the QTLs detected in the two populations studied, an analysis of synteny was carried out and the consistency of the QTLs checked over different harvests and populations. Two ester QTLs BA_6A and MH_7B, and the terpene QTL in LG3C, had been mapped in the same regions of the same LGs in both “FC50 × FD54” and “21AF” populations (Figure 3). In addition, other major QTLs have been detected that may be of interest for breeding. However, they were significant in only one of the studied populations, such as GD_3B and MH_1A detected in the “21AF,” and MH_4A and MA_7A discovered in “FC50 × FD54” (Supplementary Figure 5). To improve synteny analysis, high-dense genetic maps were used.

**Figure 4:**
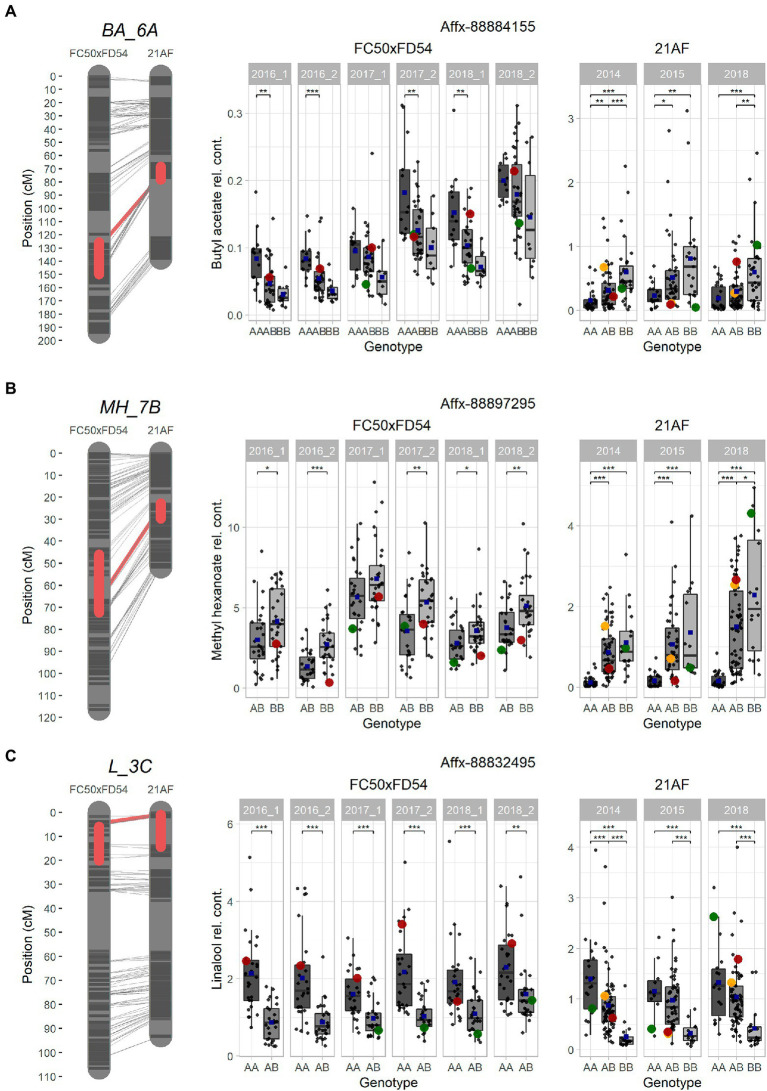
Selected major QTLs detected in the “FC50xFD54” and “21AF” maps, and boxplot of related VOCs for a common marker located in the QTL. **(A)** BA_6A QTL for acetate and octyl groups with Affx-8884155. **(B)** MH_7B QTL for methyl group with Affx-88897295. **(C)** L_3C QTL for terpene content with the Affx-88832495 marker. Red boxes on the left cover 1-LOD confidence intervals, and red line shows the SNP position in both maps. Markers colored in dark grey and chromosome in light grey. Right: Boxplot with each sample represented as a dot (progenies in grey, father line in red, mother line in green and hybrid for the “21AF” data in yellow), and the blue square indicates the average of each group. Significant levels *p* < 0.001 (***), *p* < 0.01 (**) and *p* < 0.05 (*).

Ester QTLs were spread over different LGs in both populations. Focusing on the interesting QTL cluster in LG6A, BA_6A and HA_6A detected in the “21AF” genetic map in the same region as BA_6A, O3MB_6A, OA_6A, OB_6A and OH_6A identified in the “FC50 × FD54” genetic map (Figure 3). All of these QTLs are related to acetate and octyl groups (*Acetate_6A* and *Octyl_6A*). When comparing the relative butyl acetate content in both populations, the detected content was 10-fold higher in “21AF” than in “FC50 × FD54” (Figure 4A). In the case of the Affx-8884155 marker, different segregation behaviors were observed in both populations. The homozygous allele (A) of the “FC50 × FD54” genetic map corresponded to the highest butyl acetate accumulation (*p*-value<0.001), whereas in the “21AF” genetic map, it was found to be homozygous B. The *Acetate_6A* was localized in the chromosome 6A. The smallest QTL interval was detected for the hexyl acetate in the “21AF” genetic map, which corresponds to a physical distance of 1.7Mbp and comprises 237 annotated genes (Table 2).

Different ester QTLs with a common methyl group were mapped in LG7B (*Methyl_7B*) such as MH_7B, IPH_7B and MO_7B in the “FC50 × FD54” genetic map and MH_7B and MB_7B in the “21AF” genetic map. Relative methyl hexanoate content was twice to three times higher in the “FC50 × FD54” population than in “21AF.” When observing the Affx-88897295 marker, individuals carrying the BB genotype showed a higher methyl hexanoate content than those with the AB genotypes in both populations. In fact, individuals with the AA genotype, present only in the “21AF” population, revealed an almost undetectable methyl hexanoate content (Figure 4B). Therefore, the B allele of this marker appears to be responsible for the accumulation of methyl hexanoate. The LG7B corresponded to the *F. iinumae-like* subgenome. The smallest QTL size for the *Methyl_7B* was of only 0.6 Mbp and included 93 genes (Table 2).

Two of the ester QTLs were detected only in the “21AF” population, these were MH_1A and MB_1A, both of which share a methyl group named as *Methyl_1A* (Supplementary Figure 5A). However, different QTLs related to the acetate group were detected in the same region of LG1A as the “FC50 × FD54” population. Focusing on the Affx-88812680 marker located in the *Methyl_1A* QTL interval, individuals with the homozygous A allele showed a significantly higher methyl hexanoate content than those carrying the B allele. Since the AA genotype is not present in the “FC50 × FD54” population, no significant differences were observed between “FC50 × FD54” genotypes. The *Methyl_1A* QTL covered a physical distance of 3Mbp and contained 481 annotated genes (Table 2).

Regarding the major ester QTLs involving eight different compounds related to the hexanoate group, these were all located at the end of the LG4A (Figure 3) and named as *Hexanoate_4A*. However, different stability degrees were observed in these compounds. Four of these compounds were directly related to hexanoate group (methyl, ethyl, isoamyl and hexyl hexanoates), two were related to 2-hexenoate group (methyl and ethyl 2-hexenoate), one to octanoate group (methyl octanoate) and one to decanoate group (methyl decanoate; Figure 3; Supplementary Table 9). The AB genotype of the Affx-88856759 marker in the “FC50 × FD54” population showed a significant accumulation of methyl hexanoate (*p*-value <0.001) compared to the BB genotype (Supplementary Figure 5B). But this QTL was not detected in the “21AF” population, probably masked by the effects of major QTLs in LG1A and LG7B. The *Hexanoate_4A* QTL was located at the end of LG4A which corresponds to the beginning of the described as *F. vesca-like* chromosome. The QTL interval size is 7.4Mbp containing a total of 1,233 annotated genes (Table 2). Methyl anthranilate is an aromatic ester compound providing wild strawberry aroma that was only detected in “FC50 × FD54.” Looking at the segregation of the Affx-8897207 marker located in LG7A, the AB genotype showed a significantly higher methyl anthranilate content than the AA genotype (*p*-value <0.001; Supplementary Figure 5C). This MA_7A QTL was located in the middle of the *F. vesca-like* chromosome. This QTL interval was relatively large (16.4Mbp) and therefore 2,767 genes were annotated inside (Table 2).

The terpene family, which provides floral aroma, was identified with five monoterpenes (linalool, β-pinene, D-limonene, α-terpineol and geraniol) and four sesquiterpenes (nerolidol, α-farnesene, β-farnesene and α-curcumene) in the “FC50 × FD54” population (Figure 3), and with linalool and nerolidol in the “21AF” population, since the VOC analysis was restricted to the key compounds. Different major QTLs for all these compounds were colocalized at the beginning of LG3C *Terpenes_3C* in both populations (Table 2). Focussing on the *L_3C* QTL, similar linalool relative contents were observed in both populations (Figure 4C). The genetic region of this QTL in the “21AF” genetic map showed a gap which limited the synteny to a few markers. Despite this, good synteny was observed throughout both genetic maps. Looking at the common markers inside this QTL, such as the Affx-88832495 marker, individuals with the AA genotype showed significant differences in the accumulation of linalool compared to the individuals having the AB genotype in “FC50 × FD54” or the BB genotype in “21AF.” This finding suggested that the presence of the A allele at this locus increased linalool content in both populations, while the B allele, particularly in homozygosity, appeared to correlate with a very low linalool accumulation.

Since markers from IStraw35k and 90 k arrays are not subgenome-specific, markers from the linalool QTL were located by blast analysis in all homeologous chromosomes and especially abundant in the chromosomes 3A (*F. vesca-like*) and 3C. To identify the chromosome, selected markers from the subgenome-specific 50 k SNP array, were tested in “FC50 × FD54” and those from chromosome 3C showed the same segregation as those mapped in LG3C (data not shown).

In the “FC50 × FD54” genetic map, the shortest terpene QTL interval size was observed in monoterpene compounds with an interval of 0.9Mbp and includes 171 annotated genes (Table 2). However, most of the markers from the “21AF” genetic map were mapped in the other homeologous rather than in chromosome 3C.

γ-decalactone is known to provide peach-like aroma in strawberries. In “21AF,” we found a major QTL, consistent in all harvests, in LG3B. Comparing the genotyping data of the marker Affx-88845940 with the γ-decalactone content revealed that the BB genotype correlates with a very low content of γ-decalactone and that both genotypes, AA and AB, which presented small differences between them, are related to high content of γ-decalactone. This means that A allele determines the γ-decalactone accumulation (Supplementary Figure 5D). This QTL is located at the end *F. iinumae-like* chromosome spanning a physical size of 2.4Mbp that encloses 368 annotated genes (Table 2).

### SNPs for terpene and hexanoate marker-assisted breeding

A different F1 population, called “C19,” a cross between “FC041” and “FC081” breeding lines, selected for their special aroma, composed of 46 progenies and was chosen for the study of its VOCs. The GC–MS analysis revealed that 14 out of the 19 KVCs were present in the C19 progenies.

**Figure 5:**
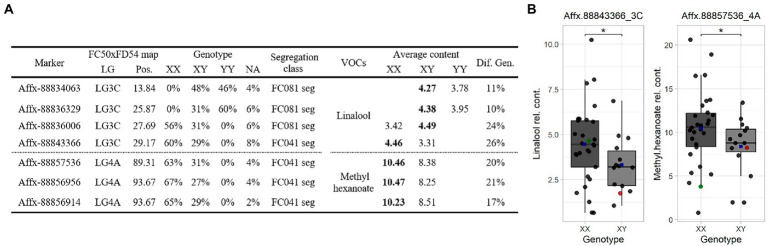
**(A)** Marker linked summary for the *Terpene_3C* and *Hexanoate_4A* QTLs in the “C19” population. Bold numbers indicate the genotype linked to the compound accumulation. **(B)** Linalool (left) and methyl hexanoate (right) boxplot for the “C19” population. Dot colors: progenies (black), “FC041” (red) and “FC081” (green). Blue squares represent means. Significant level < 0.1 (·).

SNP markers were selected for the *Terpene_3C* and *Hexanoate_4A* QTLs to construct a Fluidigm^®^ array. Hybridization array analyses showed association between four SNPs with linalool and three SNPs with methyl hexanoate accumulations. The alleles that increased linalool (Affx-88843366, *R*^2^ ≤ 26) are from the parental line “FC081” and those that increased methyl hexanoate (Affx-88856956, *R*^2^ ≤ 21) are from “FC041” (Figure 5).

## Discussion

Volatile organic compounds are key factors for fruit consumer acceptance. For this reason, breeding programs are demanding new tools that allow marker-assisted breeding (MAB) in order to satisfy the market demand. In this study, we present the aromatic profile of two strawberry populations and reveal the genome regions responsible for VOC accumulation. Additionally, some SNP markers are suggested for MAB.

The analysis of the volatilome for the “FC50 × FD54” population along six harvests identified 58 VOCs, with 16 of these being KVCs. The total number of VOCs in the “FC50 × FD54” population appears to be lower than in previous studies such as the 87 VOCs found in an F1 population (Zorrilla-Fontanesi et al., 2012), the 81 identified in a parental collection (Schwieterman et al., 2014) or the 100 discovered in a *F. vesca* NIL collection (Urrutia et al., 2017). These differences might be caused by the differences that exist between wild and cultivated strawberry, the genetic differences for VOC segregation in the used parental lines and the metabolite identification method used. In our study, Kovats retention index, used for melon (Mayobre et al., 2021), was adopted to identify volatiles, while commercial standards were used for KVCs. Ulrich et al. (2018) reviewed that between 50 and 75 compounds were identified using few strawberry accession lines as also seen in our results. More than half of the VOCs identified in our analysis were esters (57%), followed by terpenes (16%), aldehydes (12%) and lactones (5%). These proportions are comparable to those found in previous studies (Zorrilla-Fontanesi et al., 2012; Schwieterman et al., 2014; Urrutia et al., 2017).

Generally, the high correlation between compounds of the same chemical family suggests that these compounds are regulated in the same way. But also, some high correlations were observed between compounds of different chemical families. Although these correlated compounds did not necessarily share the same biosynthetic pathway, they could have some common transcription factors regulating their activity or some linked genes involved in different volatile pathways. Hence, when selecting a compound, you may be simultaneously selecting others related (Fan et al., 2021). Although the genetic factor was the main contributor to the phenotypic variance, several compounds were rather dependent on the environment.

In our analysis, a high number of QTLs (179) were discovered for 55 identified VOCs in the “FC50 × FD54” population. Among them, 94 QTLs were detected in at least three of the six harvests for 43 VOCs. This relatively low QTL stability is in concordance with previous studies in strawberry (Zorrilla-Fontanesi et al., 2012; Urrutia et al., 2017). For KVCs, 11 of the 44 discovered QTLs in the “FC50 × FD54” genetic map were detected in at least three of the six harvests for 17 different compounds. However, in the “21AF” genetic map, only nine of the 51 detected QTLs were observed in two different harvests for seven KVCs. Despite the large variation observed between seasons and populations, a good number of the detected QTLs would be valuable for breeding.

Besides, some QTLs controlling the same trait localize in different homeologous groups (HG) as observed in most of the QTL analyses in cultivated strawberry (Zorrilla-Fontanesi et al., 2011, 2012; Lerceteau-Köhler et al., 2012; Cockerton et al., 2018, 2019). In our “FC50 × FD54” population, six VOC_QTLs were mapped in HGs, with always one QTL in one HG being more stable and significant than the others. We therefore suggest that the same genes located in different HGs can contribute to the same trait, but one would have a predominant role and the others would modulate the accumulation of the VOCs (Lerceteau-Köhler et al., 2012).

Some QTLs have been validated in different genetic backgrounds. An interesting example is the *Terpenes_3C* QTL, responsible for the pleasant floral aroma, identified at the beginning of LG3C. In the “21AF” population this QTL showed higher LOD score than in the “FC50 × FD54” population. The LOD score difference is mainly due to the exclusive presence in the “21AF” population of the allele associated with a very low terpene accumulation in homozygosis (Figure 3C). Even if *Terpenes_3C* is localized in the 3C chromosome, some of its markers map to the 3B and 3D chromosome, indicating a high synteny between the homeologous chromosomes. A terpene synthase cluster or a non-specific terpene substrate protein placed in this QTL region might explain the accumulation of the terpene components. Another hypothesis is that some post-transcriptional modifications may alter the volatile terpene accumulation.

Similar to our finding, *F.* × *ananassa* nerolidol synthase, *FaNES1*, has been characterized as important for linalool and nerolidol synthesis (Aharoni et al., 2004), mapped in LGIII-4 (Zorrilla-Fontanesi et al., 2012) and annotated in 3D. Additionally, when having the functional enzyme, cultivated and wild octoploid strawberries produce higher linalool content (Chambers et al., 2012). Other nerolidol synthases, *FaNES1-like* and *FaNES2*, are annotated in 3B. These three genes show the same syntenic position as our QTL located in 3C. A very recently published genetic analysis of flavor compounds in cultivated strawberry, found also a cluster of terpene QTLs on chromosome 3C (Barbey et al., 2021). Although a non-functional allele was described in *F. vesca* (Aharoni et al., 2004; Chambers et al., 2012), terpene QTLs were also mapped in Fvb3 (Urrutia et al., 2017) and a cluster of seven *FvNES1* genes exists in the updated *F. vesca* genome annotation (Li et al., 2019). Since these results are based on the version 1.0.a1 of the cultivated strawberry genome (Edger et al., 2019) which presents high synteny between the homeologous chromosomes, mismatches may be likely to occur during assembling and therefore an homeologous *FaNES1* gene could also be located in 3C. Nevertheless, the involvement of a new gene in the accumulation of these compounds should not be discarded.

In cultivated strawberry, ester compounds are the most abundant family providing a wide range of fruity aroma. Several ester QTLs were found in different LGs such as LG1A, LG4A, LG6A and LG7B. According to our study, QTLs for different ester compounds were mapped in the same region regardless of mapping populations. In LG1A, methyl butanoate and methyl hexanoate QTLs mapped in the “21AF” genetic map while hexyl acetate, hexyl butanoate and octyl butanoate QTLs mapped in “FC50 × FD54.” These “21AF” QTLs might be related to *Methyl_1A*, whereas the “FC50 × FD54” QTLs may be related to *Hexyl_1A*. In the literature, different ester QTLs, including one for methyl hexanoate, were also mapped in LGI-1 (Zorrilla-Fontanesi et al., 2012). Other examples are the QTLs in LG6A mapped for different compounds in different populations, such as octyl group compounds, *Octyl_6A*, discovered in “FC50 × FD54” and acetate related compounds, *Acetate_6A*, in “21AF.” In previous studies, QTLs for different compounds were also found clustered together without any obvious relationship between these compounds, such as in the F1 population used by Zorrilla-Fontanesi et al. (2012) and in a multiple family studied with GWAS by Fan et al. (2021). Where the BA_6A QTL was mapped in both populations, the most significant marker shared, showed different genotypes for butyl acetate accumulation, this means that we are still quite far from the causal gene (Figure 4A). Our *Hexanoate_4A* QTL, mapped only in “FC50 × FD54,” which seems to validate the cluster of ester QTLs mapped in LGIV-1 of the “1,392 × 232” population (Zorrilla-Fontanesi et al., 2012).

QTLs for methyl hexanoate, isopropyl hexanoate and methyl octanoate were co-localized in the LG7B. These three compounds shared a methyl group, and we called the resulting QTL *Methyl_7B*. The MH_7B presented higher significance in the “21AF” compared to the “FC50 × FD54” genetic maps. This difference is due to the homozygous genotype of the allele linked to the low accumulation of methyl hexanoate that is only present in the “21AF” population (Figure 4B). The last step for ester biosynthesis is catalysed by alcohol acyl transferase (AAT). This enzyme can use a wide variety of substrates (Aharoni et al., 2000). Seven AAT genes were annotated in the *F.* × *ananassa* genome, located in LG1D (2), 7A (3), 7B (1) and 7C (1). These genes are mostly conserved in *Fragaria* genus, such as *FcAAT1* in *F. chiloensis* (Gonzalez-Aguero et al., 2009) and four AAT genes in diploid *F. vesca* genome annotated in LG6 (1) and LG7 (3) being one FvAAT enzyme already characterized (Beekwilder et al., 2004). The annotated gene encoding an AAT named as FxaC_26g18860 (Liu et al., 2021) is a candidate for our *Methyl_7B* QTL.

Methyl anthranilate, derived from the degradation of amino acids, is defined as the wild strawberry aroma which is appreciated by consumers and consequently by aroma breeding programs. In the “FC50 × FD54” population, several methyl anthranilate QTLs were mapped in different LGs, such as in LG1A, LG4C, three LGs of the HG5 and LG7A. Methyl anthranilate was not detected in the “21AF” population as this compound is known to be present in only a few cultivars (Olbricht et al., 2008; Schwieterman et al., 2014). The anthranilate acid methyl transferase gene, *FaAAMT*, mapped in the *F. vesca* chromosome 4 (Pillet et al., 2017), may be responsible of the MA_4C QTL that we detected. The HG5 and LG7A QTLs are probably related to the methyl anthranilate QTLs found in LG5 and LG7 in *F. vesca* (Urrutia et al., 2017). Markers in the region of a highly stable and major QTL, such is MA_7A, would be appropriate for MAB to select towards methyl anthranilate accumulation.

The peach aroma is attributed to γ-decalactone presence and amount. A total of six QTLs for lactone compounds were detected in the two studied populations in LG2A, LG3B, LG4A, LG6B and LG6D. The major GD_3B QTL was only detected in the “21AF” population. Although the allele linked to the high γ-decalactone accumulation was present in both populations, only the “21AF” population exhibited the low γ-decalactone accumulation genotype. This QTL is likely related to the omega fatty acid desaturase gene, *FaFAD1*, involved in the γ-decalactone synthesis and mapped in LGIII-2 (Zorrilla-Fontanesi et al., 2012; Chambers et al., 2014; Sanchez-Sevilla et al., 2014). Unfortunately, this gene is not annotated in the *F.* × *ananassa* genome due to an 8 kb deletion in non-producing γ-decalactone cultivars (Oh et al., 2021).

The “FC50 × FD54” volatilome revealed that this population is suitable for the discovery of volatile QTLs. Some of these QTLs are related to liking compounds (Schwieterman et al., 2014; Ulrich and Olbricht, 2016; Fan et al., 2021) and validate QTLs from previous studies. Still, much more effort is needed to narrow down these genomic regions in order to come across candidate genes that allow a better understanding of how these compounds accumulate and to therefore find diagnostic markers for selection in strawberry breeding.

## Data availability statement

The original contributions presented in the study are included in the article/Supplementary material, further inquiries can be directed to the corresponding author.

## Author contributions

AM and MM contributed to the conception and design of the study. PR-S performed the experimental and statistical analysis and wrote the first draft of the manuscript. All authors contributed to the article and approved the submitted version.

## Funding

PR-S was supported by a PhD grant CPD2015-0185 from MINECO (FPI-INIA). This work was supported by grant RTA2013-00010-00-00 and PID2020-119052RR-I00 from FEDER/Ministerio de Ciencia, Innovación y Universidades – Agencia Estatal de Investigación and by the CERCA Programme/Generalitat de Catalunya. We acknowledge financial support from the Spanish Ministry of Science and Innovation-State Research Agency (AEI), through the “Severo Ochoa Programme for Centres of Excellence in R&D” SEV-2015-0533 and CEX2019-000902-S.

## Conflict of interest

The authors declare that the research was conducted in the absence of any commercial or financial relationships that could be construed as a potential conflict of interest.

## Publisher’s note

All claims expressed in this article are solely those of the authors and do not necessarily represent those of their affiliated organizations, or those of the publisher, the editors and the reviewers. Any product that may be evaluated in this article, or claim that may be made by its manufacturer, is not guaranteed or endorsed by the publisher.

## Footnotes

^1^ https://webbook.nist.gov/chemistry/gc-ri/

## Supplementary material

SUPPLEMENTARY FIGURE 1 Violin plot representing VOC distributions of parents and progeny of the “FC50 × FD54” population. Harvest colors: 2016 (reddish), 2017 (orangish) and 2018 (bluish). Dots: “FC50” (green), “FD54” (red). Stars indicate KVCs.

SUPPLEMENTARY FIGURE 2 Heatmap visualization of Pearson correlation between VOCs in six harvests of the “FC50xFD54” population (*p*-value < 0.05). Stars indicate KVCs.

SUPPLEMENTARY FIGURE 3 Principal component analysis (PCA) plot in VOCs: (A) Dim1 vs Dim2 and (B) Dim1 vs Dim3. Harvest: 2016 (reddish), 2017 (orangish) and 2018 (bluish).

SUPPLEMENTARY FIGURE 4 KVC contents in the “21AF” population. A. Violin plot representing KVC distributions. Dots: “Dover” (green), “Camarosa” (red) and H-21 (yellow). B. Heatmap visualization of Pearson correlation in three years (*p*-value < 0.05). C. Heatmap representation of hierarchical clustering analyses (HCA) and D. Principal component analysis (PCA) plot in three years.

SUPPLEMENTARY FIGURE 5 QTLs of interest found in one of the two populations studied. A. *MH_1A* (Methyl hexanoate) QTL synteny and boxplot of the Affx-88812697 marker for “FC50 × FD54” and “21AF” population. B. *MH_4A* (Methyl hexanoate) QTL synteny for “FC50 × FD54” and “21AF” population and boxplot of the Affx-88856759 marker. **C**. *MA_7A* (Methyl anthranilate) QTL synteny for “FC50xFD54” and “21AF” population and boxplot of the Affx-88897207 marker. D. *GD_3B* (γ-decalactone) QTL synteny and boxplot of the Affx-88845940 marker for “FC50 × FD54” and “21AF” population. Left: red boxes cover 1-LOD confidence intervals and red line is the Affx-88845940 position. Marker class represented as codominant (green), “FC50” segregating (blue) and “FD54” segregating (yellow). Right: Boxplot with each sample represented as dots and the blue square the average of each group. Significant levels <0.001 (***), <0.01 (**) and <0.05 (*).
