# Supplementary figures and images for: Inheritance of esters and other volatile compounds responsible for the fruity aroma in strawberry
Source: Front Plant Sci. 2022 Aug 12;13:959155. doi: 10.3389/fpls.2022.959155 (PMC9412188; doi:10.3389/fpls.2022.959155)

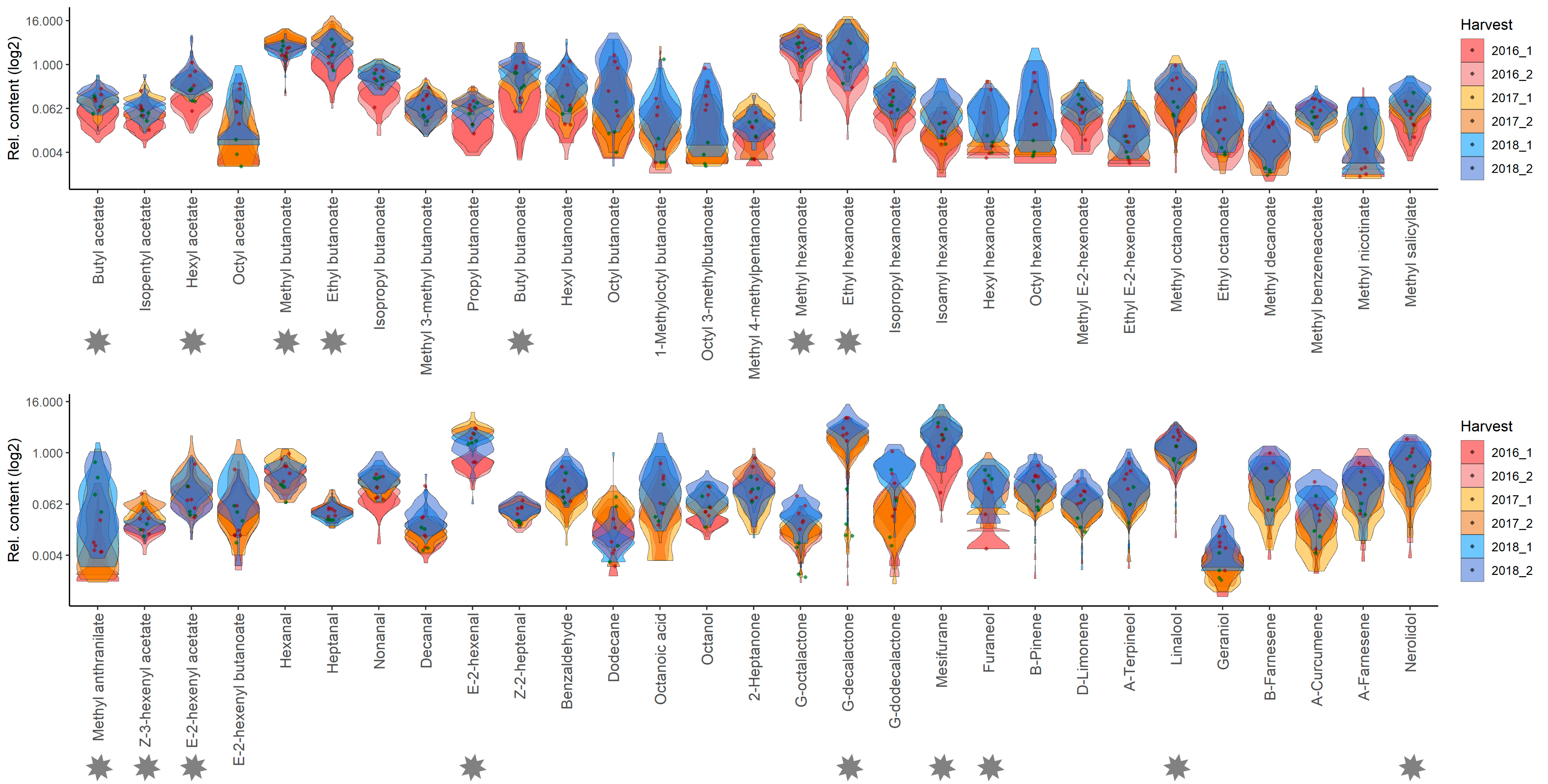

Supplement: SUPPLEMENTARY FIGURE 1 — Violin plot representing VOC distributions of parents and progeny of the “FC50 × FD54” population. Harvest colors: 2016 (reddish), 2017 (orangish) and 2018 (bluish). Dots: “FC50” (green), “FD54” (red). Stars indicate KVCs. [file Image_1.JPEG]

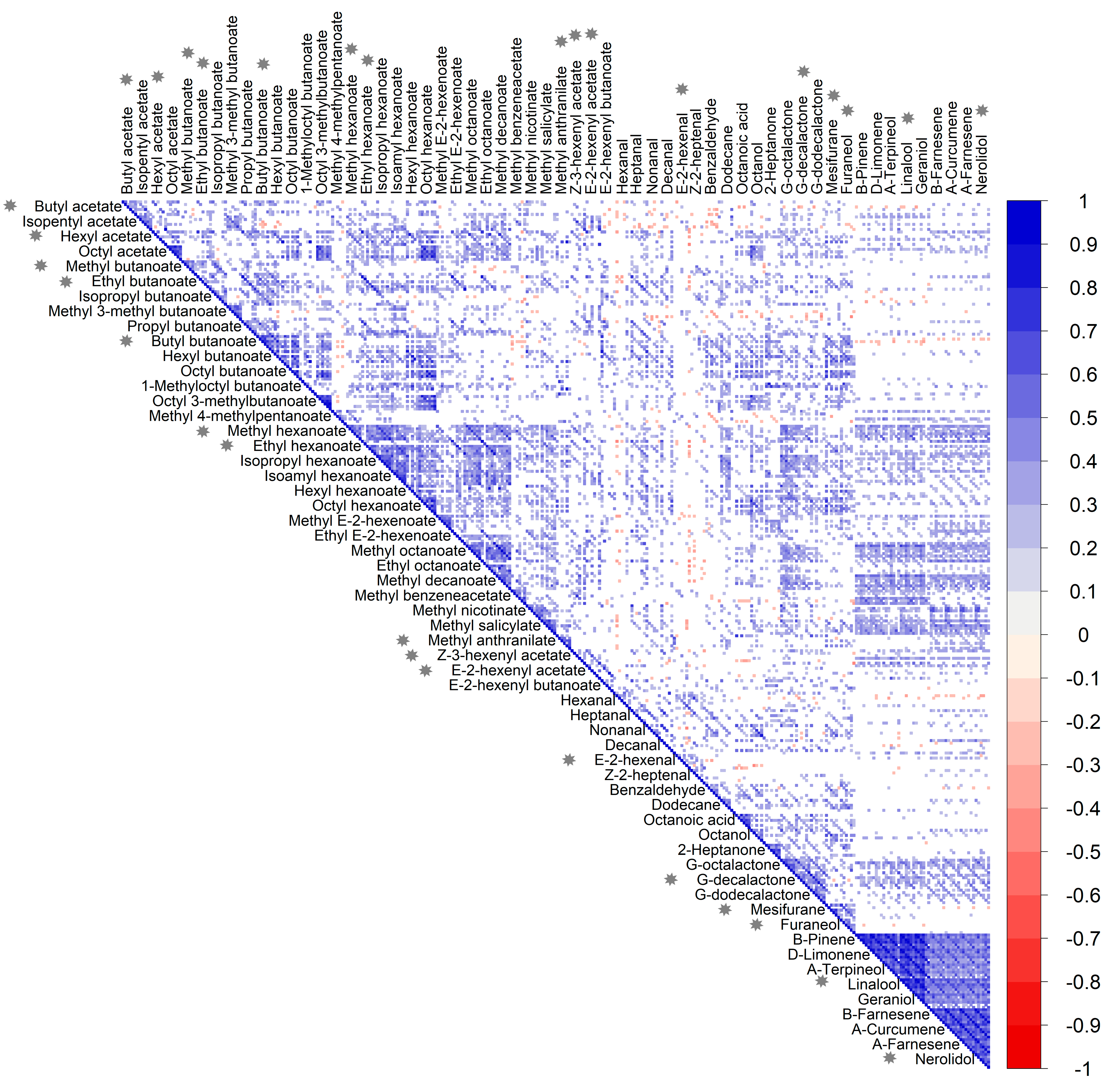

Supplement: SUPPLEMENTARY FIGURE 2 — Heatmap visualization of Pearson correlation between VOCs in six harvests of the “FC50xFD54” population (p-value < 0.05). Stars indicate KVCs. [file Image_2.JPEG]

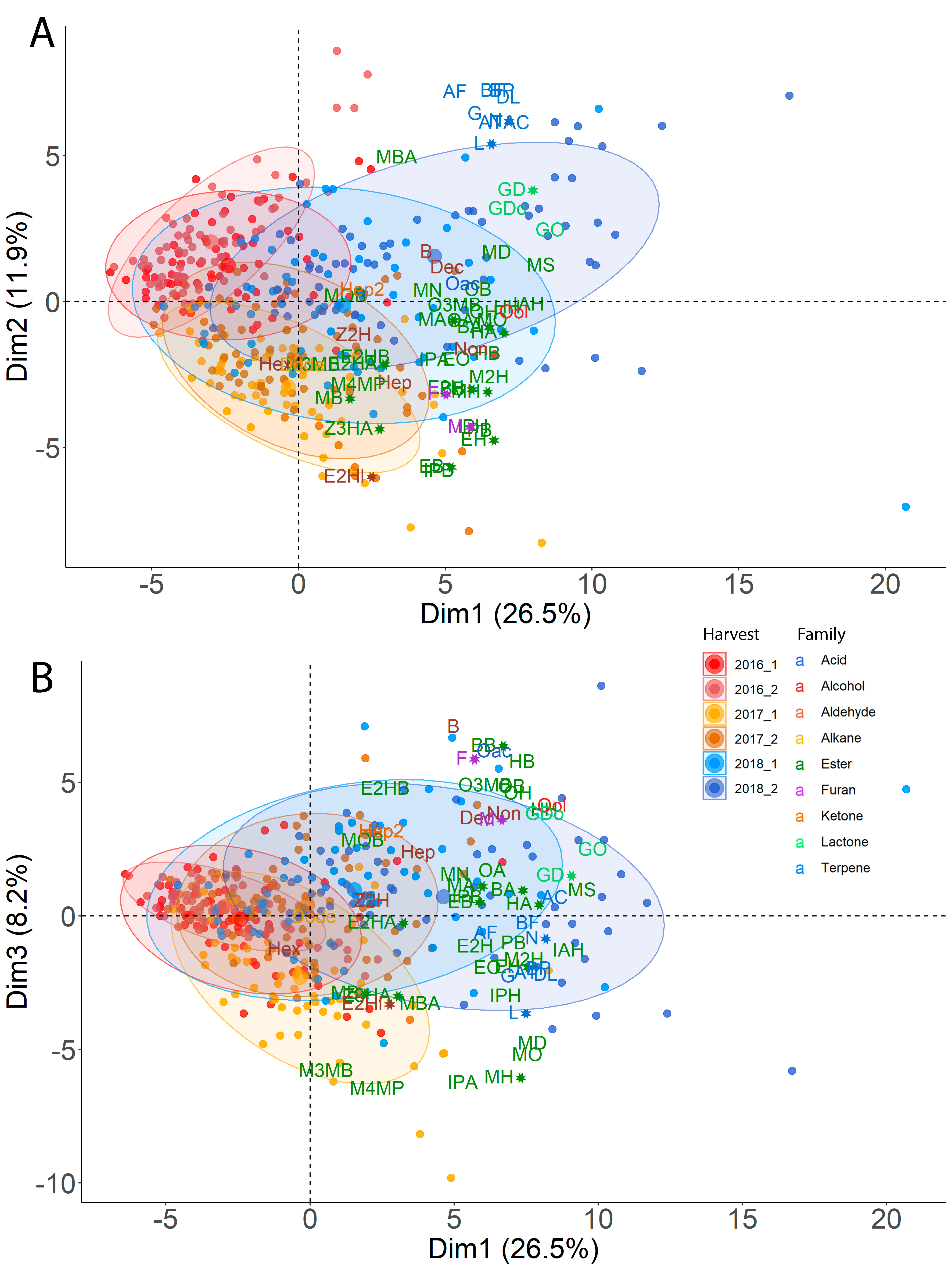

Supplement: SUPPLEMENTARY FIGURE 3 — Principal component analysis (PCA) plot in VOCs: (A) Dim1 vs Dim2 and (B) Dim1 vs Dim3. Harvest: 2016 (reddish), 2017 (orangish) and 2018 (bluish). [file Image_3.JPEG]

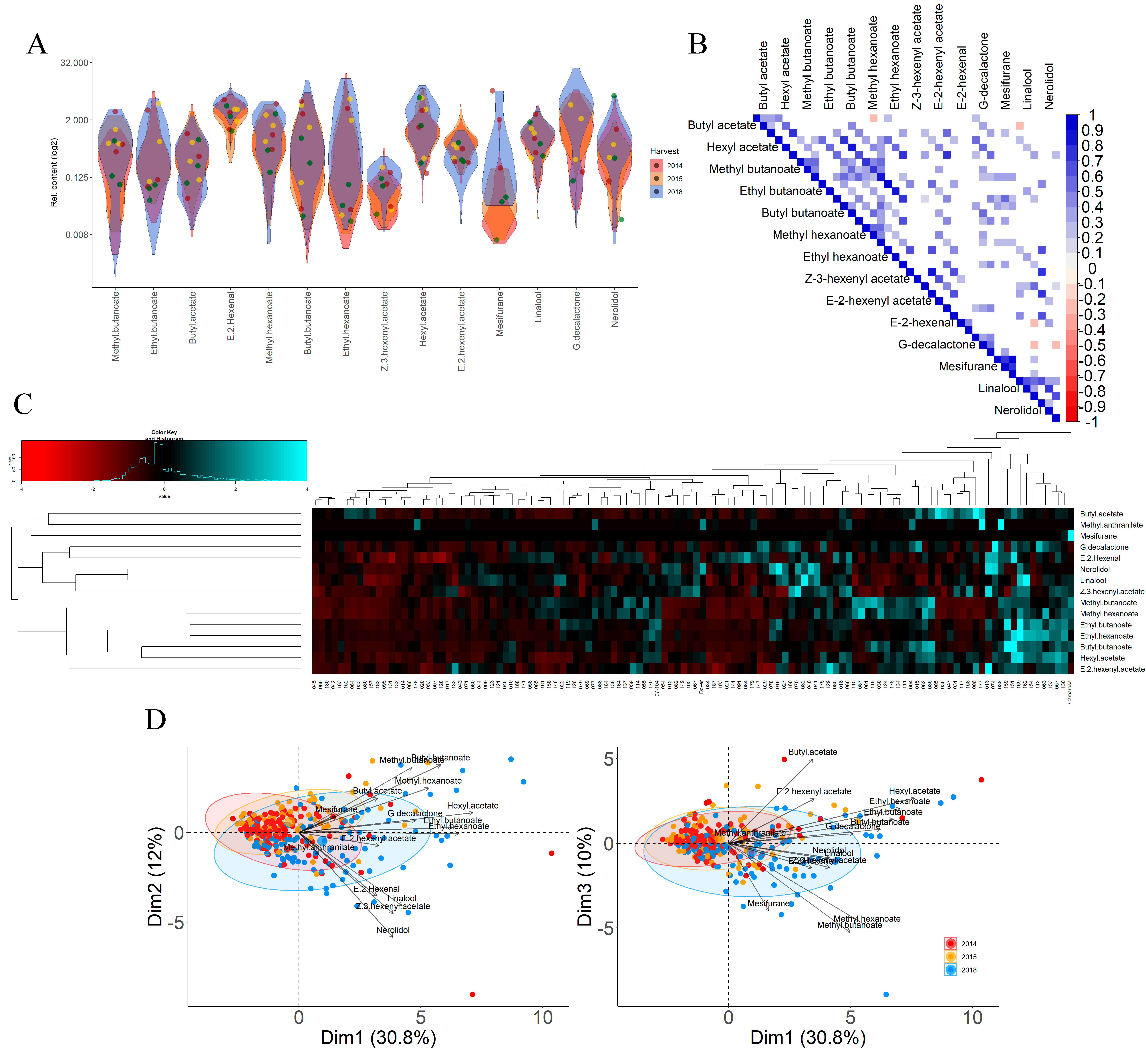

Supplement: SUPPLEMENTARY FIGURE 4 — KVC contents in the “21AF” population. A. Violin plot representing KVC distributions. Dots: “Dover” (green), “Camarosa” (red) and H-21 (yellow). B. Heatmap visualization of Pearson correlation in three years (p-value < 0.05). C. Heatmap representation of hierarchical clustering analyses (HCA) and D. Principal component analysis (PCA) plot in three years. [file Image_4.JPEG]

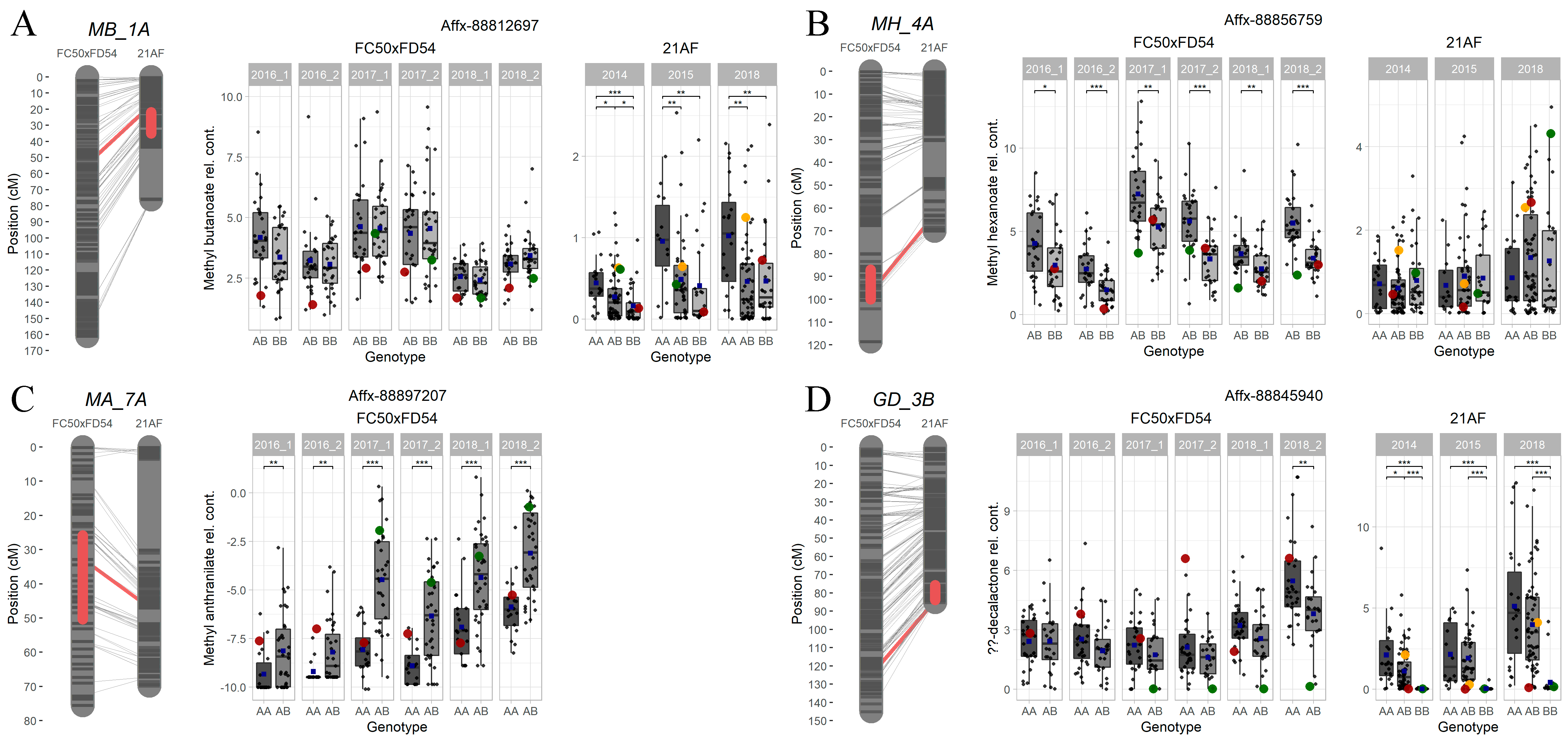

Supplement: SUPPLEMENTARY FIGURE 5 — QTLs of interest found in one of the two populations studied. A. MH_1A (Methyl hexanoate) QTL synteny and boxplot of the Affx-88812697 marker for “FC50 × FD54” and “21AF” population. B. MH_4A (Methyl hexanoate) QTL synteny for “FC50 × FD54” and “21AF” population and boxplot of the Affx-88856759 marker. C. MA_7A (Methyl anthranilate) QTL synteny for “FC50xFD54” and “21AF” population and boxplot of the Affx-88897207 marker. D. GD_3B (γ-decalactone) QTL synteny and boxplot of the Affx-88845940 marker for “FC50 × FD54” and “21AF” population. Left: red boxes cover 1-LOD confidence intervals and red line is the Affx-88845940 position. Marker class represented as codominant (green), “FC50” segregating (blue) and “FD54” segregating (yellow). Right: Boxplot with each sample represented as dots and the blue square the average of each group. Significant levels <0.001 (***), <0.01 (**) and <0.05 (*). [file Image_5.JPEG]
